# Poplar Autophagy Receptor NBR1 Enhances Salt Stress Tolerance by Regulating Selective Autophagy and Antioxidant System

**DOI:** 10.3389/fpls.2020.568411

**Published:** 2021-01-20

**Authors:** Wanlong Su, Yu Bao, Yingying Lu, Fang He, Shu Wang, Dongli Wang, Xiaoqian Yu, Weilun Yin, Xinli Xia, Chao Liu

**Affiliations:** ^1^Beijing Advanced Innovation Center for Tree Breeding by Molecular Design, Beijing Forestry University, Beijing, China; ^2^National Engineering Laboratory for Tree Breeding, Beijing Forestry University, Beijing, China; ^3^College of Biological Sciences and Technology, Beijing Forestry University, Beijing, China

**Keywords:** salt stress, selective autophagy, poplars, NBR1, antioxidant system activity, ubiquitinated proteins

## Abstract

Salt stress is an adverse environmental factor for plant growth and development. Under salt stress, plants can activate the selective autophagy pathway to alleviate stress. However, the regulatory mechanism of selective autophagy in response to salt stress remains largely unclear. Here, we report that the selective autophagy receptor PagNBR1 (*neighbor of BRCA1*) is induced by salt stress in *Populus*. Overexpression of PagNBR1 in poplar enhanced salt stress tolerance. Compared with wild type (WT) plants, the transgenic lines exhibited higher antioxidant enzyme activity, less reactive oxygen species (ROS), and higher net photosynthesis rates under salt stress. Furthermore, co-localization and yeast two-hybrid analysis revealed that *PagNBR1* was localized in the autophagosome and could interact with ATG8 (autophagy-related gene). *PagNBR1* transgenic poplars formed more autophagosomes and exhibited higher expression of *ATG8*, resulting in less accumulation of insoluble protein and insoluble ubiquitinated protein compared to WT under salt stress. The accumulation of insoluble protein and insoluble ubiquitinated protein was similar under the treatment of ConA in WT and transgenic lines. In summary, our results imply that *PagNBR1* is an important selective autophagy receptor in poplar and confers salt tolerance by accelerating antioxidant system activity and autophagy activity. Moreover, the *NBR1* gene is an important potential molecular target for improving stress resistance in trees.

## Introduction

Soil salinity is an adverse environmental factor, which has a serious impact on plant growth, development, and productivity (Zorb et al., 2005). About 6% of the world’s total land area and nearly 20% of irrigated land are exposed to excessive salt concentrations, and the problem is becoming progressively worse (Munns and Tester, 2008). Therefore, understanding the mechanism of plant tolerance and breeding salt-tolerant plants is of great biological significance for the amelioration and utilization of salinated soil. Salt stress is triggered by excessive amounts of sodium (Na^+^) and chloride ions in soil (Cl^−^; Ismail et al., 2014), which puts plants under multi-component stress, including ion stress, osmotic stress, and oxidative stress (Munns and Tester, 2008; Cabot et al., 2014). Osmotic, ionic, and oxidative stress can induce serious damage, which includes the disruption of cellular structures and macromolecules (e.g., proteins, DNA, and lipids; Wang et al., 2009; Golldack et al., 2014; Genisel et al., 2015). An important strategy for plants to cope with salt stress is to maintain the water potential and K^+^/Na^+^ homeostasis by synthesizing several classes of osmolytes (e.g., proline, polyamine, and sugar alcohol) and regulating the activity of ion channel proteins, such as SOS1 (Deinlein et al., 2014; Yang and Guo, 2018). Another important strategy is scavenging reactive oxygen species through the antioxidant system, such as superoxide dismutase (SOD), catalase (CAT), peroxidase (POD), and ascorbate-glutathione (AsA-GSH) cycle (Raja et al., 2017; Yang and Guo, 2018).

In recent years, autophagy, which means “self-eating,” has become an important strategy for eukaryotes in resisting stress conditions (Avin-Wittenberg et al., 2012; Avin-Wittenberg, 2019). Autophagy can be classified into different types, macro-autophagy, micro-autophagy (Cvt), and chaperone-mediated autophagy (CMA). The autophagy-related genes (ATG) discovered in yeast have been identified and studied in other eukaryotes (Cebollero and Reggiori, 2009; Wen and Klionsky, 2016), implying that autophagy is an evolutionarily conserved process in eukaryotes.

Compared with the selective CMA and Cvt pathways, the most important role of the macro-autophagy pathway is initially considered to degrade bulk cytoplasm through the non-selective pathway. Recent research has revealed that selective autophagy mechanisms are mediated by specific cargo receptor proteins (Ichimura et al., 2008; Johansen and Lamark, 2011). Several receptors have been identified in mammalian organisms, such as nuclear dot protein 52 kDa (NDP52; von Muhlinen et al., 2010), optineurin (OPTN; Liu et al., 2014), sequestosome-1 (P62/SQSTM1; Ichimura et al., 2008), and neighbor of BRCA1 gene 1 (NBR1; Kirkin et al., 2009). p62/SQSTM1 is an important selective receptor in human and mammalian cells. Previous research has largely focused on human p62/SQSTM1 (Lamark et al., 2009; Lee et al., 2017). The p62/SQSTM1 protein mainly binds and facilitates the clearance of ubiquitinated proteins that accumulate in various chronic, toxic, and degenerative diseases (Liu et al., 2016; Carroll et al., 2018). The p62/SQSTM1 protein also participates in ROS cleaning processes in human cells. NBR1 was also found in numerous metazoan species for the degradation of ubiquitinated proteins that share similar domain architecture to p62/SQSTM1 (Kirkin et al., 2009; Kenific and Debnath, 2016). In addition, both proteins are involved in the degradation of ubiquitinated proteins *via* PB1-dependent polymerization. However, the PB1 domain in p62/SQSTM1 and NBR1 differs. The PB1 domain in the p62/SQSTM1 protein harbors two OPCA (OPR/PC/AID) motifs and can oligomerize with itself; meanwhile, the NBR1 protein harbors only one OPCA (OPR/PC/AID) motif and binds with p62/SQSTM1 in a PB1-PB1 fashion (Kirkin et al., 2009; Mizushima, 2015).

Autophagy studies in plants benefit from the functional conservation of ATG proteins and autophagy has been shown to participate in multiple biological processes in plants, which include biotic stress, abiotic stress, growth, and development (Inoue et al., 2006; Zhou et al., 2015; Luo et al., 2017). In *Arabidopsis*, *atg* mutants exhibit early senescence and chlorosis phenotype, as well as nitrogen hypersensitivity or carbon nutrient deficiency (Masclaux-Daubresse, 2016). Autophagy also regulates salicylic acid signaling to inhibit programmed cell death and senescence under pathogen infection (Yoshimoto et al., 2009). Thus, autophagy is a functionally important process for plants in response to stress. Although studies on autophagy in plants have provided great progress in recent years (Li et al., 2015; Luo et al., 2017), our knowledge of selective autophagy remains limited. and the adapter of selective autophagy still needs further research.

Plant NBR1 (neighbor of BRCA1), which is a selective receptor, is homologous with mammalian p62/SQSTM1 and NBR1 proteins (Svenning et al., 2011; Zientara-Rytter et al., 2011). They share conserved domains such as the N-terminal PB1 (Phlox and Bem1), ZZ-type zinc finger domain, LIR (LC3-interacting region) motif, and C-terminal UBA (ubiquitin-associated) domain (Svenning et al., 2011). In contrast to p62/SQSTM1, the plant NBR1 protein contains four specific tryptophan (FW) residues, which are highly conserved in the homologs of NBR1 proteins (Svenning et al., 2011). Research indicates that the homopolymerized characterization of NBR1 protein in plants was similar to p62/SQSTM1, thereby suggesting that plant NBR1 possesses hybrid properties of mammalian NBR1 and p62/SQSTM1 proteins (Svenning et al., 2011; Zientara-Rytter and Sirko, 2014). Meanwhile, p62/SQSTM1 protein is confined to metazoans, and only NBR1 protein is detected throughout the plant kingdom. In *Arabidopsis*, catalases were highly aggregated proteins in the *nbr1* mutant suggesting the ital roles of NBR1 in the cleaning of ROS (Zhou et al., 2014). Thus, this study speculated that the plant NBR1 could be involved in the degradation of ubiquitinated insoluble proteins and the cleaning of ROS under stress conditions.

*Populus* is one of the main forestation tree species that exhibits fast growth, adaptability, and worldwide distribution. This species has important economic and ecologic value for the quick recovery of vegetation and soil remediation (Yannikos et al., 2014; Radojčić Redovniković et al., 2017). Soil salinity has seriously affected the growth of trees and timber yields. *Populus* is a model plant for tree research (Tuskan et al., 2006), and understanding the mechanisms of salt stress resistance in *Populus* is of practical significance for the amelioration and utilization of salinated soil.

Selective autophagy mechanism in response to abiotic stress remains unreported in *Populus*. However, the homologs of selective autophagy receptors were found in *Arabidopsis* (AtNBR1), tobacco (Joka2/NBR1), and tomato (NBR1). Previous research has focused on heat stress (Zhou et al., 2013), chilling stress (Chi et al., 2020), and sulfur deficiency (Zientara-Rytter et al., 2011). Only a few studies have emphasized the response of NBR1-mediated selective autophagy to salt stress in plants, and the functional mechanism of plant NBR1 is still largely unclear. Our previous studies showed that expression levels of the *NBR1* gene in *Populus* is induced by salt stress and oxidative stress. In the current study, we hypothesized that the poplar NBR1 protein may function in response to salt stress through the degradation of ubiquitinated proteins and activation of antioxidant enzymes. To explore this we isolated an *NBR1* gene from Poplar 84 K clone (*Populus alba* × *P. tremula* var. *glandulosa*; *PagNBR1*), a hybrid poplar introduced from Korea that has been extensively cultivated in northern China (Qiu et al., 2019). Transgenic poplars were generated to evaluate the potential tolerance mechanism mediated by the *NBR1* gene in response to salt stress.

## Experimental Procedures

### Plant Material and Treatments

Poplar 84 K hybrids (*Populus alba* × *P. tremula* var. *glandulosa*) have been commonly used in molecular biological studies (He et al., 2018). The 84 K saplings were approximately 5 cm high culturing on the rooting medium. They were transplanted in seed pots (10 cm × 10 cm), in the phytotron artificial climate chamber with a 16/8-h day/night cycle (22–25°C, 60% humidity, 54 μmol m^−2^ s^−1^) in Beijing Forestry University, Beijing, China. The plants were watered with a nutrient solution every 3 days for 60 days before treatment.

The expression level of *PagNBR1* was analyzed in different tissues of poplar 84 K including roots (R), xylem (X), phloem (P), mature leaves (ML), old leaves (OL), and young leaves. For salt stress, 84 K saplings were watered with 400 mM NaCl solution. For oxidative stress, 100 μM MV (Sigma, 856177) or 0.5 mM H_2_O_2_ solution was sprayed once on the leaves of 84 K. For plant hormone treatment, the 84 K saplings were sprayed once on leaves with a solution containing 100 μM abscisic acid (ABA, SigmaA1049), 5 mM ethephon (ET, Sigma 45473), 2 mM salicylic acid (SA, SigmaS5922), 100 μM Methyl Jasmonate (MeJA, Sigma, 392707) or 500 μM Gibberellin (GA3, Sigma 48870). For the control groups, 84 K saplings were watered or sprayed with water. Mature leaves (from 7th to 11th) were collected after 0 h, 1 h, 2 h, 4 h, 6 h, 12 h, 24 h of treatment and immediately put into liquid nitrogen and stored at −80°C for use.

### RNA Extraction and qRT-PCR Analysis

Total RNA was extracted from the Control and treatment materials using the cetyltrimethylammonium bromide (CTAB) method (He et al., 2018). Total RNA quality and integrity were detected using the NanoDrop 2000 spectrophotometer (Thermal Fisher Scientific, West Palm Beach, FL) and agarose gel electrophoresis. 2 μg RNA was used for reverse transcription using the Prime Script™ RT reagent Kit with gDNA Eraser (Perfect Real Time, Takara, RR047Q) following the manufacturer’s guidance. The qRT-PCR was performed as previously methods (He et al., 2018) using the TB Green® *Premix Ex Taq*™ GC (Perfect Real Time, Takara, RR071Q). The relative expression level of genes was calculated using 2^-∆∆Ct^ as described previously (Wang et al., 2014, 2015b). Primers were designed using Primer 5.0 (Sigma-Aldrich Corp., St. Louis, MO) and listed in Supplementary Table S1. Three biological replicates and four technical replicates were carried out in the experiment.

### Isolation and Bio-Informatics Analysis of *PagNBR1*

The complete coding sequence (CDS) of *PagNBR1* was amplified using high-fidelity thermostable DNA polymerize Prime Star (Takara, R045A). The primer used in this study is listed in Supplementary Table S1. The multiple sequence alignment for the proteins of AtNBR1, PtrNBR1, and PagNBR1 were performed with DNAMAN V6. The conserved domains were analyzed using the conserved domain database.^1^ The physicochemical property of PagNBR1 protein was predicted using ProtParam database.^2^

### Subcellular Location of *PagNBR1*

To confirm the subcellular localization of the PagNBR1 protein, the *PagNBR1* and *AtATG8a* genes were inserted into a plant binary expression vector *35S-GFP* which was reconstructed from *pCAMBIA1300-GUS* under the control of CaMV*35S* promoter. The constructed vectors *35S::PagNBR1::GFP* and *35S::mCherry::AtATG8a* were transiently transformed into tobacco protoplast using the methods previously described (Yoo et al., 2007; Chupeau et al., 2013). The *35S::mCherry::AtATG8a* was used as an autophagosome marker and constructed as previous methods (Ren et al., 2014). The *35S-GFP* was used as vector control. The signal of GFP and mCherry were detected using a confocal microscope (Leica TCS SP8; Leica, Wetzlar, Germany) after being transformed in tobacco protoplast for12-16 h. The excitation wavelength was 488 nm (GFP), 580 nm (mCherry), and the emission wavelengths 507 nm (GFP) and 610 nm (mCherry) were used to detected fluorescence signal.

### Yeast Two-Hybrid Analysis

To evaluate the interactions of PagNBR1 and ATG8 proteins in 84 K (PagATG8), a yeast two-hybrid system was prepared. The CDS of *PagNBR1* and *PagATG8* (*ATG8a*, *ATG8e*, *ATG8g*, *ATG8h*) genes were inserted into pGBKT7 and pGADT7 vectors to generate *BK-PagNBR1* and *AD-PagATG8s* vectors, respectively. Then the vectors were co-transformed into yeast competent AH109 cells. The *AD/BK*, *AD/BK-NBR1*, *BK/AD-ATG8s* were performed as a negative control. The positive transformants were selected on the SD/-Leu-Trp medium and then serial dilution assay including 1, 1/10, 1/100, and 1/1000 was performed on SD/-Trp-Leu-His-Ade medium at 30°C for 4–6 d.

### Generation of Transgenic Plants

The CDS of *PagNBR1* was inserted into the *35S*-*GFP* vector before the *GFP* with termination codon deleted under the control of the CaMV35S promoter. The fusion recombinant vector was transformed into *Agrobacterium tumefactions strain EHA105* before infecting poplar leaves. The Agrobacterium-mediated transformation was performed using previously described methods (He et al., 2018) and the detailed information was supported in the Supplement Methods.

Total DNA was isolated using the CTAB method (He et al., 2018) and materials were collected from the saplings selected using hygromycin B. PCR was performed using 20 bp 35S promoter as the forward primer and 21 bp *GFP* as reserve primer to validate the transgenic plants at the DNA level. qRT-PCR was also used to analyze the expression level of the *PagNBR1* gene in wild type (WT) and transgenic lines. The reference gene *UBQ* was selected based on a previous result (Wang et al., 2015b). The primers used are listed in Supplementary Table S1.

### Assessment of Salt Stress Tolerance in Transgenic Lines

Twenty-day asexual-propagation seedlings were transplanted to seedling pots (10 cm length × 10 cm width × 10 cm height) with the same seedling substrate (turfy soil: vermiculite =1:1) and then the culture in a phytotron artificial climate chamber with a16/8-h day/night cycle (25°C, 60% humidity, 54 μmol m^−2^ s^−1^). To assess the tolerance of salt stress at the whole-plant level, 60-day WT and transgenic lines were subjected to 400 mM NaCl solution until damaged spots were visible on the leaves of the plants. For the control group, WT and transgenic lines were cultured in the same conditions but replaced the NaCl solution with water. After NaCl treatment, the plants were harvested for enzyme activity, relative water content, MDA content, Na^+^ content, K^+^ content Fresh weight (FW), and Dry weight (DW) shoots and roots. The treatment was performed with four biological replicates and three times technological replicates, respectively. The detailed treatment methods were listed in the Supplement Methods.

To further verify whether autophagy is involved in the process of the degradation of insoluble protein and the insoluble ubiquitinated proteins among WT and transgenic lines under salt stress, the leaves of WT and transgenic lines were prepared in small rounds and treated with water (Control group), 200 mM NaCl and 200 mM NaCl with 0.5 μM Concanamycin A (ConA, Sigma-Aldrich, United States) and harvested after 12 h of treatment.

### Malonaldehyde Concentration, Electrolyte Leakage, and Relative Water Content

Malonaldehyde (MDA) concentration and Electrolyte leakage (EL) were analyzed in WT and transgenic plants after treatment. MDA was measured using thiobarbituric acid (TBA)-reactive substances (Li et al., 2012). Electrolyte leakage was measured according to a previous protocol (Shi et al., 2013). In brief, nine leaf discs with 0.5 cm diameter were immersed in 10 ml deionized water for 8 h for detecting initial conductivity (C_i_). Then, the samples were incubated at 95°C for 30 min to destroy the leaf tissues and release the whole electrolyte. After cooling the solution to room temperature, the max conductivity (C_max_) was detected. The EL was estimated as the formula EL(%) = (C_i_/C_max_) × 100%.

Relative water content (RWC) was also analyzed in WT and transgenic plants after treatment. In brief, the fresh weight of leaves was recorded as FW. Then, the samples were incubated in distilled water for 24 h to measure the saturated weight (WW). In the end, they were drying at 80°C and dry weights were measured. The relative water content was estimated following the formula RWC (%) = (FW-DW)/(WW-DW) × 100%. 27 replicates containing nine biological and three technical replicates were conducted per experiment.

### Measurement of Photosynthetic Gas Exchange and Chlorophyll Fluorescence Parameters

The Li-6400 Photosynthesis System (Li-Cor, Lincoln, NE, United States) was used in this study. Fully expanded mature leaves (from 7th to 11th) were selected to detect the net photosynthetic rate (Pn), Transpiration rate (Tr), stomatal conductance (Cond), and intercellular CO_2_ concentration (Ci) with 400 μmol/l CO_2_ concentration and 800 μmol m^−2^ s^−1^ photon flux density. For Quantum yield of photochemical energy conversion in PSII (Y (II)) and non-photochemical Quenching (qN), the same leaves were detected with a Dual-PAM-100 (Walz Heinz GmbH, Effeltrich, Germany).

### Determination of Na^+^ and K^+^ Content

Root, stem, and leaves were collected from WT and transgenic poplars after treatment. They were heated at 120°C for 15 min and kept at 80°C until constant weight. Then the materials were ground into powder for the determination of Na^+^ and K^+^. 0.2 g materials were digested with concentrated sulfuric acid and titrated with H_2_O_2_. Na^+^ and K^+^ contents were determined with an atomic absorption spectrophotometer as described previously (Yang et al., 2015).

### Measurement of ROS Accumulation, Antioxidant System Activity

Poplar 84 K leaves (0.2 g) were ground in liquid nitrogen and an ice-cold phosphate buffer solution (PBS, pH 7.8) was used to extract antioxidant enzyme contain SOD, CAT, POD. The homogenate was centrifuged, and the supernatant was used to measure antioxidant enzyme activity.

SOD activity was measured using the inhibition of photochemical nitro blue tetrazolium reduction methods (He et al., 2018). POD activity was measured by detecting the oxidation of guaiacol (He et al., 2018). CAT activity was measured using the ultraviolet absorption method by determining the decrease of H_2_O_2_ (Patra et al., 1978).

For the detection of O_2_^−^ and H_2_O_2_ accumulation, histochemical staining methods were performed by immersing the leaves in nitro blue tetrazolium (NBT, Sigma, 11585029001) and diaminobenzidine (DAB, Sigma, D7679) respectively. The content of H_2_O_2_ in leaves was detected by monitoring ferrous ion oxidation using xylenol orange as an indicator (Zhang et al., 2017).

The content of AsA, DHA, GSH, and GSSG was analyzed according to the method described by Wang et al. (2012).

### Isolation of Soluble, Insoluble Proteins, and Insoluble Ubiquitinated Proteins

The poplar 84 K leaves collected from the control and NaCl treatment groups were prepared. The samples were homogenized in protein extraction buffer (100 mM Tris/HCl, pH 8.0, 10 mM NaCl, 1 mM EDTA, 1% Triton X-100, and 0.2% ß-mercaptoethanol). Soluble and insoluble proteins were isolated by low-speed centrifugation as previously described (Zhou et al., 2013). The contents of soluble and insoluble proteins were examined using Brilliant Blue G-250 dye (Bradford, 1976). Ubiquitinated protein in insoluble protein was detected using Plant ubiquitin (Ub) ELISA Kit (Shanghai renjie biotechnology co., ltd, Cat. No RJ21777^3^) according to the manufacturer’s instructions. In brief, purified plant Ub antibody was used to coat microtiter plate wells and to make a solid-phase antibody, then Ub solution was added to the wells. It was combined with an antibody labeled with HRP and became an antibody-antigen-enzyme-antibody complex. After washing completely, a TMB substrate solution was added to the microtiter plate wells. The TMB substrate then turned a blue color under the catalyzation of the HRP enzyme. The reaction was terminated by a sulfuric acid solution and the color change was measured spectrophotometrically at a wavelength of 450 nm. The concentration of Ub in the samples was then determined by comparing the O.D. of the samples to the standard curve.

### Autophagy Analysis

To analyze whether autophagy was induced in WT and transgenic lines under salt stress, leaves were excised and immediately immersed with 100 μM MDC (Sigma-Aldrich, 30432) for 30 min and then washed with phosphate-buffered saline. The fluorescence signal was detected at 400-508 nm according to Wang et al. (2015a).

TEM analysis was performed following previous methods. After 4 days of NaCl treatment, the mature leaves excised from poplar 84 K were used for observation. Small strips of leaves (1 mm × 2 mm, 10 pieces from each plant) were immediately mixed with 2.5% glutaraldehyde and fixed under dark conditions at 4°C for 12 h. PBS was used to wash the pieces before being fixed in 1% osmium tetroxide. Subsequently, different concentration gradients of ethanol (30–100%) were used. Fixed materials were packaged with Epon812. The ultrathin section was prepared using an ultra-microtome (Leica ULTRACUT, Wetzlar, Germany). Serial sections were examined using a JEOL-1230 transmission electron microscope (Hitachi, Tokyo, Japan) for autophagosome observation.

### Statistics

All data were subjected to Statistical Product and Service Solutions v. 17.0 (SPSS, Chicago, IL) for statistical analysis. LSD multiple range tests were used to detect the significant differences between individual means. Differences at the 5% level were considered significant and denoted by lowercase letters or asterisk among different groups.

## Results

### Molecular Characterization of *PagNBR1*

**Figure 1:**
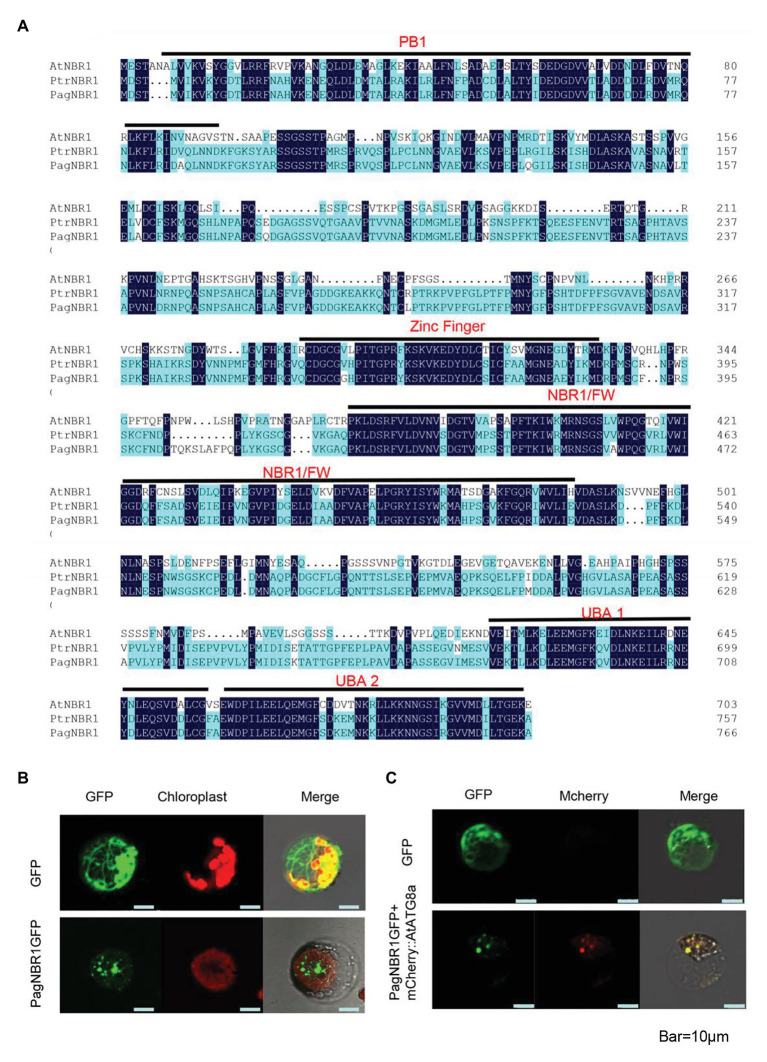
Sequence alignments of NBR1 in *Arabidopsis thaliana* and poplars and subcellular location of PagNBR1. **(A)** Sequence alignments of AtNBR1, PtrNBR1, and PagNBR1. Identical amino acids are shown with a dark blue background. The domains contained in sequences are shown with a black line and label. **(B)** Confocal laser scanning microscopy images of poplar mesophyll protoplast that transiently expresses PagNBR1:GFP and GFP **(C)** Confocal laser scanning microscopy images of poplar mesophyll protoplast that transiently expresses PagNBR1::GFP and mCherry::AtATG8a in tobacco protoplast. Bar = 10 um.

NBR1, a selective autophagy receptor protein, is conserved in both animal and plant kingdoms. The target gene identified from Poplar 84 K contains 2301 bases which encode 767 amino acids. Protein primary structure analysis showed that the molecular weight of the protein is 83.5 KDa, and its theoretical pI is 4.94. Multiple sequence alignment analysis revealed that the protein sequence shares 42.1% identity with AtNBR1 protein and 96.6% identify with NBR1 protein from *P. trichocarpa* (PtrNBR1). The protein contains an FW domain (or named NBR1 domain), which is also highly conserved in homologs of NBR1 proteins. The N-terminal PB1 domain, ZZ-type zinc finger, and C-terminal UBA-domain (Figure 1A and Supplementary Figure S1) were also found in the protein, and all the three domains are highly conserved in the mammalian proteins of p62/SQSTM1 and NBR1 and plant NBR1 protein (Svenning et al., 2011). These results indicated that the protein is highly homologous with AtNBR1 protein suggesting that they may have a similar biological function. Thus, we named it PagNBR1 protein.

### PaNBR1 Localizes in Autophagosome

Previous studies have demonstrated that NBR1 functions as an autophagy receptor, which facilitates specific substrate degradation by autophagy (Deosaran et al., 2013; Zhou et al., 2014). To confirm the detailed subcellular location of PagNBR1, we transiently transformed the recombinant vector *35S: NBR1: GFP* into tobacco protoplast (Figure 1B). AtATG8a protein is used as an autophagosome-localized marker (Yoshimoto et al., 2004) and the *mCherry::AtATG8a*, which is restricted in our research, was co-transformed with *PagNBR1::GFP* to confirm its targeting position. Figures 1B,C show that the green fluorescence of PagNBR1 was localized in punctuated cytosolic bodies, and the red fluorescence of AtATG8a was localized in punctuated cytosolic bodies with overlapping of green fluorescence. This suggests that the PagNBR1 protein is located in the autophagosomes.

### Expression Patterns of *PagNBR1* in Different Tissues and in Response to Abiotic Stress

**Figure 2:**
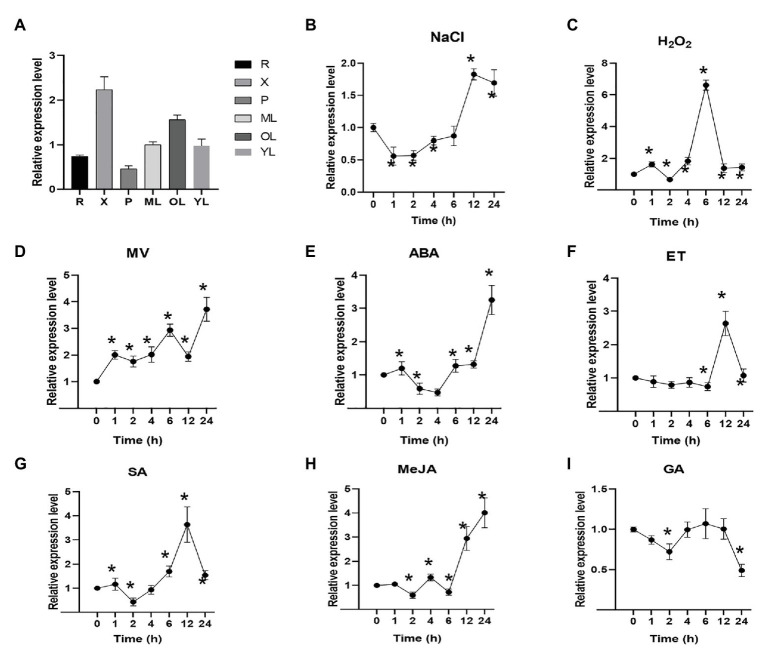
*PagNBR1* expression patterns in different tissues and under different treatments. **(A)** Relative *PagNBR1* expression levels in different tissues; Relative *PagNBR1* expression levels in response to different treatment, NaCl **(B)**, H_2_O_2_ **(C)**, MV **(D)**, ABA **(E)**, ET **(F)**, SA **(G)**, Me-JA **(H)**, and GA **(I)**. R, root; YL, young leaves; X, xylem; P, phloem; ML, Mature leaf; OL, old leaf. Total RNA was isolated from leaf samples collected at indicated times. Each treatment was performed with three biological replicates (*n* = 3) and values are means ± SE. Asterisks indicate that values were significantly different from the control at *p* < 0.05.

To illuminate the potential roles of the *PagNBR1* gene, the expression patterns in different tissues, including the root (R), xylem (X), phloem (P), young leaf (YL), mature leaf (ML), and old leaf (OL), were analyzed by qRT-PCR under normal conditions. The accumulation of *PagNBR1* was relatively high in the xylem, mature leaves, and old leaves (Figure 2A). In addition, the expression levels of PagNBR1 increased with leafage adding among young leaves, mature leaves, and old leaves implying that it may play important roles during plant senescence.

Plant hormones are involved in many processes of plant development and stress tolerance (Verma et al., 2016). To illuminate whether *PagNBR1* were induced by different plant hormones, the plant hormones such as ABA, ET, Me-JA, SA, and GA3, were sprayed on the leaves of WT plants. *PagNBR1* gene showed similar expression patterns after ABA and Me-JA treatment (Figures 2E,H). It was downregulated by 0.5-fold after 2 (ABA) or 4 h (Me-JA) of treatment and upregulated by three-fold to four-fold after 24 h of treatment. In the case of ET treatment, the expression of the *PagNBR1* gene was largely induced two-fold to three-fold after 6–12 h of treatment. The expression level of *PagNBR1* reached a peak after 12 h treatment of ET (Figure 2F). SA treatment downregulated the transcription level of *PagNBR1* during the first 2 h and was induced by more than three-fold of the control after 12 h of treatment (Figure 2G). GA treatment downregulated the expression levels of PagNBR1 after 2 h and 24 h of treatment, but it was similar after 4 to 12 h of treatment (Figure 2I).

To further analyze the expression levels of *PagNBR1* during different stress conditions, WT plants were treated with NaCl, MV, and H_2_O_2_. Materials were collected after 0, 1, 2, 4, 6, 12, and 24 h. Our results showed that *PagNBR1* mRNA level decreased to the minimum value after 1 h of salt treatment and then increased after 2 h of treatment and peaked to about two-fold of the control after 12 h of treatment (Figure 2B). H_2_O_2_ treatment dramatically increased the transcription level of the *PagNBR1* gene by more than six-fold within 6 h of treatment and subsequently decreased and recovered to the basal level from 12 to 24 h (Figure 2C). MV treatment exhibited different expression patterns at an early stage (i.e., 0–2 h) with NaCl and H_2_O_2_. The *PagNBR1* mRNA level increased with MV treatment duration and it was induced by more than three-fold of the control after 24 h of treatment (Figure 2D). In summary, our results showed that salt stress, oxidative stress, and plant hormones (which included ABA, ET, Me-JA, and SA) could induce *PagNBR1* gene expression suggesting *PagNBR1* maybe play important roles in resisting different external environmental stress.

### *PagNBR1* Overexpression Improves Salt Stress Tolerance in Poplar

**Figure 3:**
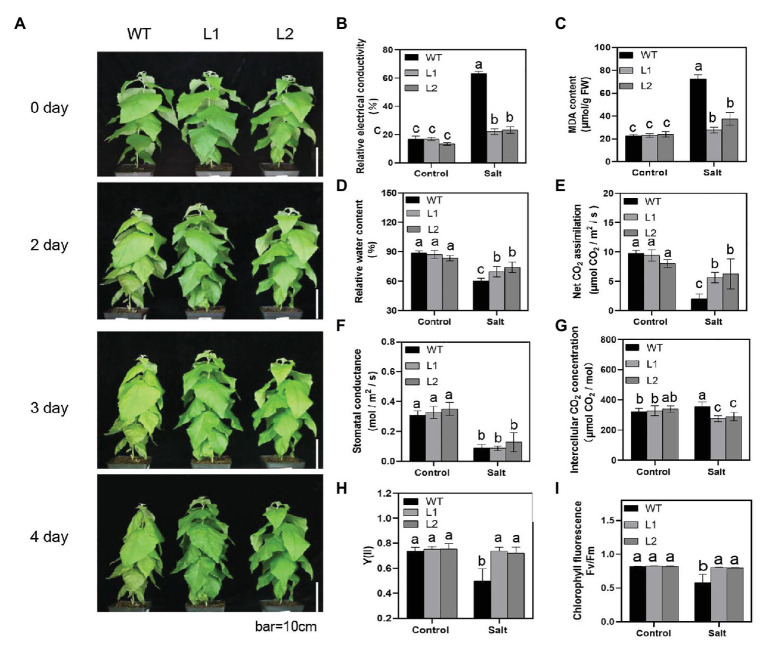
*PagNBR1* overexpression enhances salt tolerance in transgenic poplars. **(A)** Effects of salt stress on the growth of wild-type (WT) plants and overexpressed transgenic plants (L1 and L2). About 60-day-old plants were treated with 0 or 400 mM NaCl for 4 days and photographed before and after treatment. Bar = 10 cm; **(B)** Relative electrical conductivity. **(C)** MDA content. **(D)** Relative water content (RWC). **(E)** Net CO_2_ assimilation. **(F)** Stomatal conductance. **(G)** Intercellular CO_2_ concentration. **(H)** Quantum yield of photochemical energy conversion in PSII (Y [II]). **(I)** Fv/Fm. These data were measured after 4 days of treatment. Each treatment was performed with three biological replicates (*n* = 3) and values are means ± SE. Data were analyzed through LSD multiple range tests in the ANOVA program of SPSS (IBM SPSS17.0). Different letters indicate significant differences between treatments at a level of *p <* 0.05.

Abiotic stress such as salt stress, oxidative stress, and plant hormone treatment promoted the expression of the *PagNBR1* gene (Figure 2). Thus, we hypothesized that *PagNBR1* may be involved in plant tolerance to salt stress. Transgenic poplar 84 K was generated and identified at the DNA and transcription levels (Supplementary Figure S2). Before formal experiments for collecting data, we performed a pre-experiment to calibrate the concentration of NaCl solution using two-month-old WT and transgenic lines. Results showed that the leaves of WT or transgenic lines did not exhibit obvious phenotype and wilted after 10 days of treatment with 200 mM NaCl (Supplementary Figure S4). This might be because watering the NaCl solution more than once could cause the enrichment of NaCl. The leaves of WT wilted after 4 days of treatment with 400 mM NaClD. During the treatment process, we watered the seedlings only once. We used 400 mM of NaCl solution and used it to simulate salt stress conditions. The seedlings treated with 400 mM NaCl were used in the treatment group, whereas the control group was replaced with water. The leaves of WT were more wilted than those of transgenic poplars, which suggested that WT suffered more serious damage than transgenic poplars after treatment (Figure 3A).

The potential and permeability of the cell membrane are sensitive to stress conditions. An increase in membrane lipid peroxidation and membrane permeability results in increased EL and MDA. Thus, EL and MDA are important indicators that reflect the integrity of the cell membrane under stress conditions. To evaluate the degree of membrane damage, we detected the EL and MDA levels in WT and transgenic plants under control and salt stress conditions (Figures 3B,C). Compared with the control group, salt stress significantly increased the MDA level in WT and transgenic poplars. After 4 days of NaCl treatment, the MDA content in transgenic plants increased from 23 to 23.7 μmol/g FW to 27.7–37.3 μmol/g FW. However, WT plants showed a more than three-fold increase from 22.5 μmol/g FW to 72.4 nmol/g FW (Figure 3C). Moreover, the EL increased between WT and transgenic plants after salt stress. The WT plants exhibited a three-fold increase from 16.8 to 63.3%, whereas transgenic poplar leaves presented a one-fold increase from 13.4–16.9% to 22.1–23% under salt stress (Figure 3B). The relative water content (RWC) also showed lower values in WT than transgenic plants after treatment (Figure 3D). The results of FW/DW (Supplementary Figure S6) supported the RWC results. In summary, these results indicated that overexpression of *PagNBR1* enhanced tolerance of salt stress in transgenic lines.

### Photosynthetic Gas Exchange and Chlorophyll Fluorescence Parameters

To analyze the effect of *PagNBR1* on the characteristics of photosynthetic physiology during salt stress conditions, we determined the photosynthetic parameters using the Lico-6,400 Portable Photosynthesis System. Under normal conditions, the net photosynthetic rates were not observably different between WT and transgenic poplars (Figure 3E). However, a significant difference was found between WT and transgenic poplars after salt stress (Figure 3E). After salt stress, the photosynthetic capacity of WT was about 10% of that in the control group. However, the transgenic poplars declined to nearly 50% of that in the control group (Figure 3E). Similarly, the inter-cellular CO_2_ concentration showed a dramatic decline in transgenic plants under stress conditions but not in WT (Figure 3G). The variation trend of stomatal conductance showed no notable difference between WT and transgenic plants under stress conditions (Figure 3F).

Chlorophyll fluorescence is an indicator of the photosynthetic efficiency of plants (An et al., 2014). Y(II) is an indicator of the conversion efficiency of PSII, and the FV/Fm presents the maximum optical quantum yield of PSII which reflects the potential maximum light energy conversion efficiency in plants (Alpuerto et al., 2016). Therefore, Y(II) and Fv/Fm were measured using PAM100. Salt stress was found to cause a decrease in Y(II) from 0.75 to 0.49 in WT plants. However, no significant decrease was observed in transgenic poplars (Figure 3H). Salt stress decreased Fv/Fm levels in WT. Compared with transgenic poplars, WT had a lower value of Fv/Fm under salt stress (Figure 3I). In general, these results showed that transgenic poplars had better tolerance to salt stress than WT.

### *PagNBR1* Regulates the Distribution of Na^+^ and K^+^ in Different Tissues

**Figure 4:**
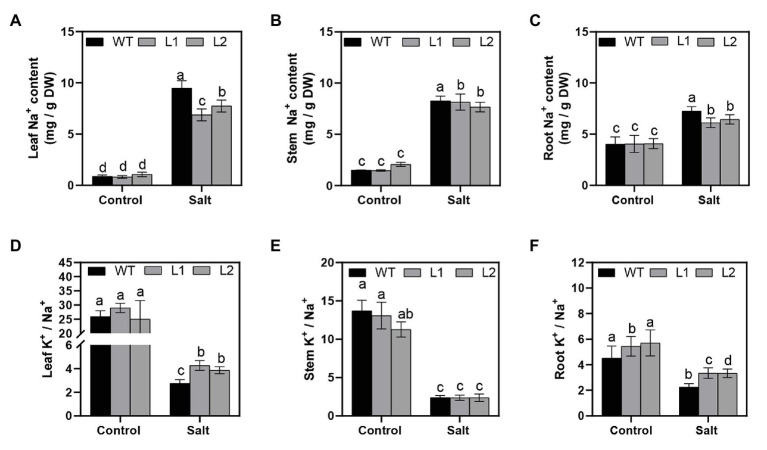
Na^+^ and K^+^ contents in different tissues of wild-type (WT) and transgenic plants (L1 and L2). After NaCl treatment, plant materials were collected and divided into leaves, stems, and roots for the estimation of Na^+^ and K^+^ contents. **(A)** Na^+^ contents of leaves, **(B)** Na^+^ contents of stems, **(C)** Na^+^ contents of roots, **(D)** K^+^/Na^+^ of leaves, **(E)** K^+^/Na^+^ of stems, and **(F)** K^+^/Na^+^ of roots. These data were measured after 4 days of treatment. Values are means (±SE) of three independent experiments (*n* = 3). Data were analyzed through LSD multiple range tests in the ANOVA program of SPSS (IBM SPSS17.0). Different letters indicate significant differences between treatments at a level of *p* < 0.05.

To explain the underlying mechanism of salt tolerance caused by *PagNBR1* through the regulation of Na^+^ and K^+^ homeostasis, the Na^+^ and K^+^ distribution in different tissues (such as roots, stems, and leaves) were measured. Under NaCl stress, the Na^+^ concentrations dramatically increased in WT and transgenic poplars (Figures 4A–C). Compared with transgenic lines, the Na^+^ concentration in leaves of WT increased observably higher about ten-fold after 4 days of treatment (Figure 4A). The Na^+^ concentration in roots of WT also increased and was observably higher than transgenic lines, but there was no significant difference in stems among WT and transgenic lines after 4 days of treatment (Figures 4B,C). Under salt stress, the dynamic balance of K^+^/Na^+^ contributed to maintaining ion balance and promoting plant development. Figures 4D–F show that all plants maintained a high K^+^/Na^+^ ratio in leaves, stems, and roots under normal conditions. The K^+^/Na^+^ ratio between WT and transgenic lines was not remarkably different in leaves in the control group (Figure 4D). Under salt stress, the K^+^/Na^+^ ratio in transgenic poplars was significantly higher than that in WT (Figure 4D). In stems, overexpressed PagNBR1 did not significantly change the ratio of K+/Na + under normal and stress conditions (Figure 4E). In roots, the K+/Na + ratio was higher in transgenic poplars than in WT under stress conditions (Figure 4F). To further illuminate the role of *PagNBR1* under salt stress, we analyzed SOS1 expression (Supplementary Figure S3). The SOS1 expression levels in the treatment group were upregulated, but the transgenic poplars exhibited 1.8-fold higher expression than the WT at the transcription level (Supplementary Figure S3). Thus, overexpression of *PagNBR1* enhanced tolerance of salt tolerance by regulating redistribution of Na^+^ and K^+^ under salt stress.

### *PagNBR1* Enhances Antioxidant System Activity Under Salt Stress

**Figure 5:**
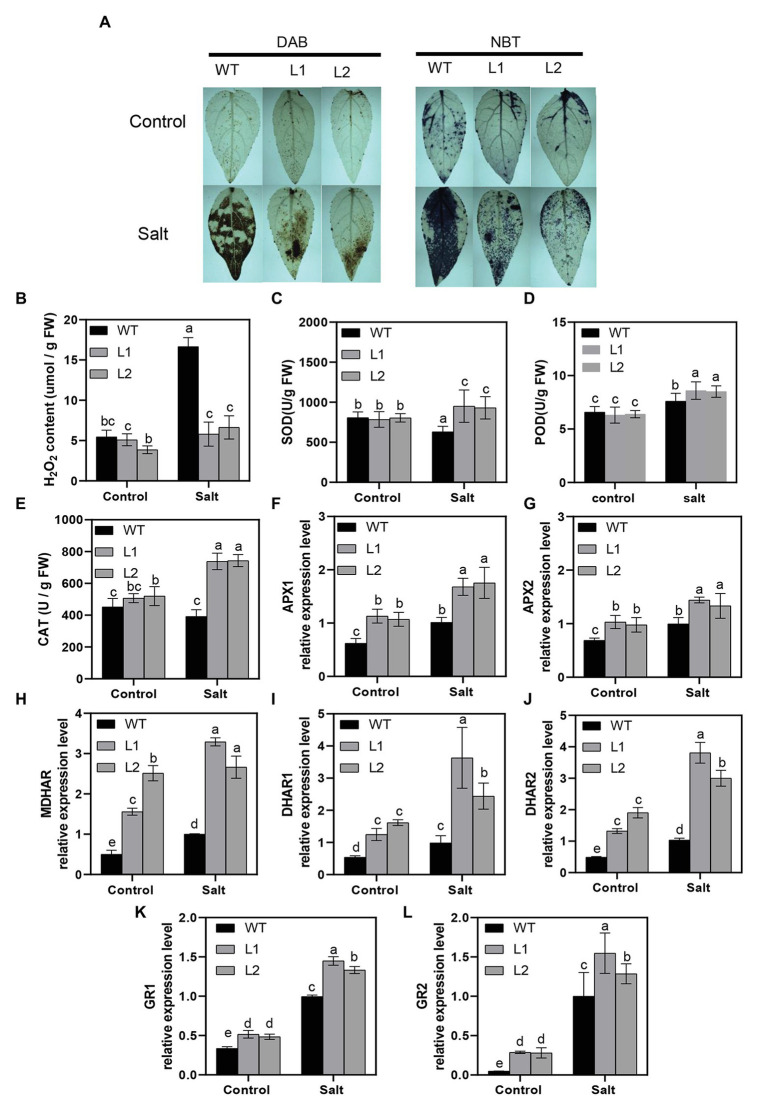
Activity of H_2_O_2_, O_2_^−^, and antioxidant system in WT and transgenic plants. **(A)** Histochemical staining with DAB and NBT for the detection of H_2_O_2_ and O_2_^−^, respectively, in WT transgenic plants before and after treatment. **(B)** Accumulation of H_2_O_2_ in WT and transgenic plants, **(C)** SOD activity, **(D)** POD activity, **(E)** CAT activity, **(F,G)** relative expression of *APXs*, **(H)** relative expression of *MDHAR*, **(I,J)** relative expression of *DHARs*, and **(K,L)** relative expression of *GRs* in WT and transgenic plants (L1, L2) under control and treatment conditions. For H_2_O_2_ and O_2_^−^, leaves were excised from lines after 4 days of treatment and immediately immersed in DAB (H_2_O_2_) and NBT(O_2_^−^). Data were measured after 4 days of treatment. Values are means (±SE) of three independent experiments (*n* = 3). Data were analyzed through LSD multiple range tests in the ANOVA program of SPSS (IBM SPSS17.0). Different letters indicate significant differences between treatments at a level of *p* < 0.05.

Reactive oxygen species (ROS) can be induced under stress conditions and play a major role in plants under abiotic stress (Choudhury et al., 2013). In the above analysis, we realized that salt stress resulted in more serious cellular membrane damage in WT than in transgenic lines (Figures 3B,C), which suggested that WT plants accumulated more ROS than transgenic poplars under salt stress. Therefore, 3,3′-diaminobenzidine (DAB) and nitroblue tetrazolium (NBT) was used to detect H_2_O_2_ and O_2_^−^ *in situ*, respectively (Figures 5A,B). Figure 4A shows that WT greatly enriched more H_2_O_2_ and O_2_^−^ than transgenic plants during salt stress. We also measured the H_2_O_2_ content by monitoring ferrous ion oxidation using xylenol orange. The results were consistent with those *in situ* in WT and transgenic poplars (Figure 5B). SOD, POD, and CAT play main roles in scavenging ROS and contribute to maintaining ROS homeostasis in plants (Xing et al., 2018; He et al., 2019). After 4 days of salt stress, the enzyme activities of SOD, POD, and CAT in transgenic lines were dramatically higher than those in WT. However, the enzyme activities between WT and transgenic lines were similar under normal conditions (Figures 5C–E).

Ascorbic acid (AsA) and glutathione (GSH) cycling are important factors in scavenging H_2_O_2_ and O_2_^−^ for plants to main ROS homeostasis under stress conditions (Wang et al., 2012; Sun et al., 2018a,b). To further reveal the function of AsA-GSH cycling in scavenging ROS, we detected the crucial genes *APX*, *MDHAR*, *DHAR*, and *GR* at the transcription levels in WT and transgenic lines. As shown in Figures 5F–L, the transcription level of these genes increased under salt stress conditions in WT and transgenic poplars; however, transgenic poplars demonstrated significantly higher transcription levels compared with WT. Similar results were obtained in the control group (Figures 5F–L). We also analyzed the content of AsA + DHA and GSH + GSSG and results showed that transgenic poplars kept higher levels at metabolic levels than that of WT poplars (Supplementary Figure S6). In general, PagNBR1 could enhance tolerance of salt stress *via* activating the antioxidant system.

### *PagNBR1* Can Interact With *PagATG8s*

**Figure 6:**
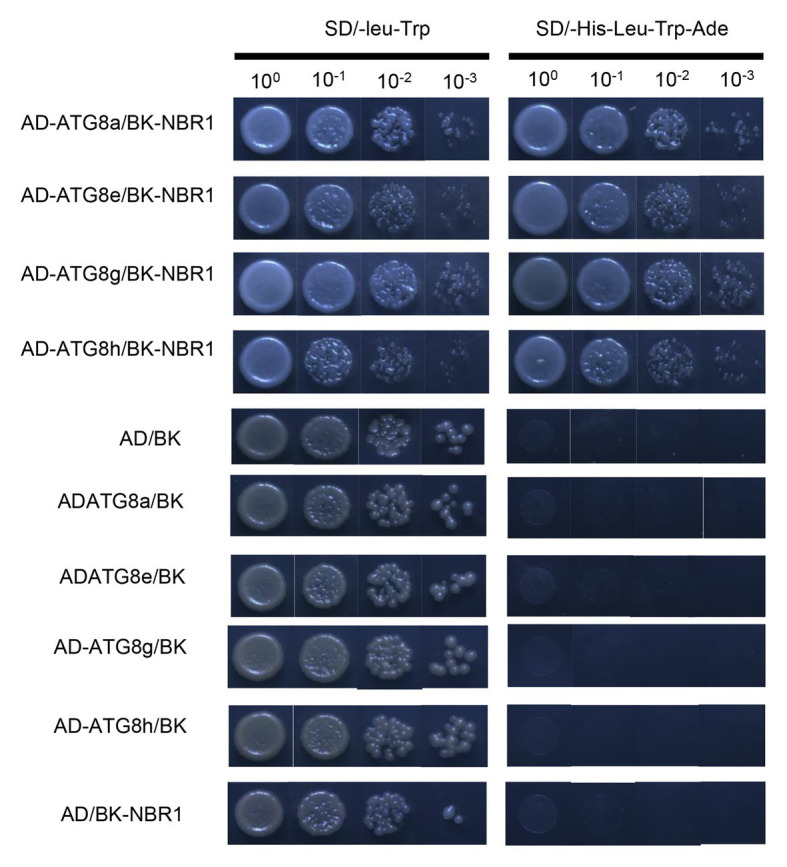
Interaction analysis of PagNBR1 and ATG8s using a yeast two-hybrid system. pGBK-NBR1 co-transformation with pGAD-ATG8a, pGAD-ATG8e, pGAD-ATG8h, pGAD-ATG8g, and pGAD-T7. Negative control of the interaction of PagNBR1 and ATG8s. Co-transformation of pGAD-T7/pGBK-T7, pGBK-T7/pGAD-ATG8a/8e/8g/8h, and pGADT7/PGBK-NBR1. All plasmids were transformed into yeast AH109 competent cells and cultured on SD/-leu-Trp and SD/-Ade-His-Leu-Trp medium. Data are from three independent experiments (three yeast lines measured in each independent experiment). One representative image of them is shown.

Previous studies have revealed that the AtNBR1 protein can interact with the AtATG8 protein to facilitate the degradation of insoluble ubiquitinated protein aggregates through selective autophagy (Zhou et al., 2013). In the present study, to identify a similar interaction between the proteins of PagNBR1 and PagATG8, we performed a two-hybrid experiment in yeast. *PagATG8* (*ATG8a ATG8e ATG8h ATG8g*) was fused with *pGADT7*, and *PagNBR1* was fused with *pGBKT7*. The recombination vector *pGBK-NBR1* and *pGAD-ATG8* were co-expressed in yeast AH109 cells. A negative control was also prepared (*pGBK-NBR1*/*pGADT7*; *pGBKT7/pGAD-ATG8s*). Figure 6 shows that the yeast containing *pGBK-NBR1* and *pGAD-ATG8* grew normally on SD/−leu-Trp and SD/-Ade-His-Leu-Trp medium. In the negative control, normally grown yeast was observed on SD/-Leu-Trp, and no yeast was observed on SD/-Ade-His-Leu-Trp medium. Notably, our results showed that PagNBR1 could interact with PagATG8, which suggested that PagNBR1 is a receptor and may have functional characteristics similar to AtNBR1.

### *PagNBR1* Promotes the Autophagic Clearance of Ubiquitinated Protein Aggregates

**Figure 7:**
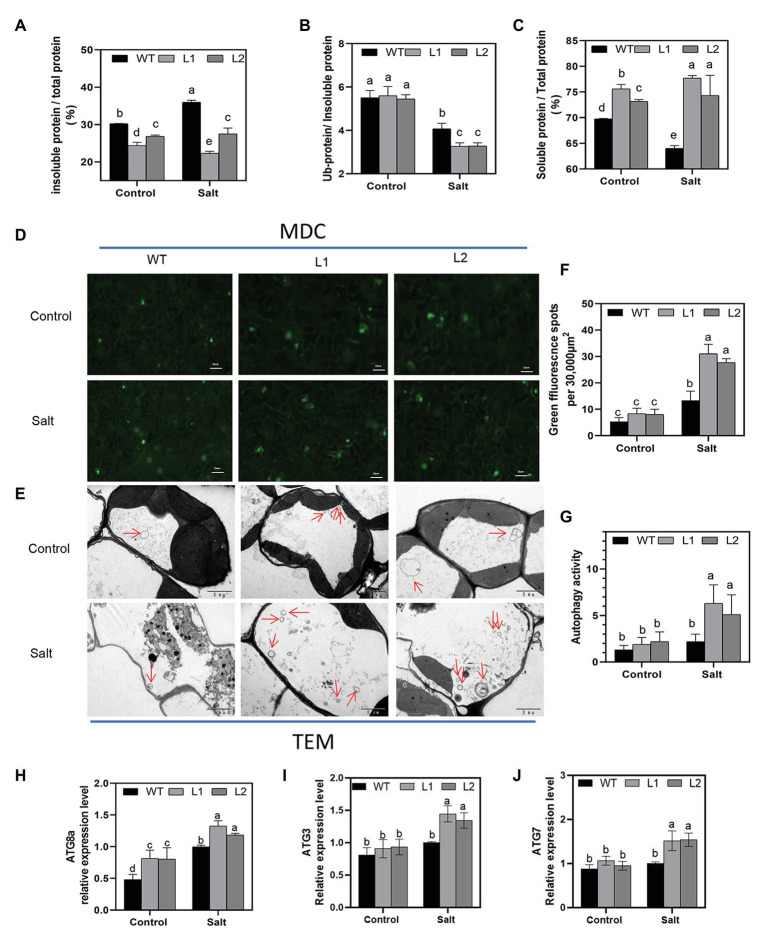
Accumulation of insoluble protein, insoluble ubiquitinated protein, the autophagosome, and *ATG* expression levels in WT and transgenic plants under control and treatment conditions. **(A)** Proportion of insoluble protein in total protein, **(B)** proportion of ubiquitinated protein in insoluble protein and soluble protein in total protein, **(C)** proportion of soluble protein in total protein, **(D)** MDC-stained autophagosomes are shown in green fluorescence signal (bar = 25um), **(E)** representative TEM images of autophagosome in WT and transgenic plant leaves, **(F)** number of green fluorescence signal per 30,000um^2^ area in **(D)**, **(G)** quantified autophagosomes in **(E)** and **(H–J)** relative expression of *ATG8a, AT3*, and *ATG7*. More than 10 cells were used to quantify autophagic structures in mesophyll cells in the TEM results. These data were measured after 4 days of treatment. Values are means (±SE) of three independent experiments (*n* = 3). Data were analyzed through LSD multiple range tests in the ANOVA program of SPSS (IBM SPSS17.0). Different letters indicate significant differences between treatments at a level of *p* < 0.05.

NBR1 is considered a kind of selective autophagy receptor to clear ubiquitinated protein aggregates and ub-tagged organelle *via* interacting with ATG8 in plants and mammals (Svenning et al., 2011; Jung et al., 2019). Our results indicated that the proteins of PagNBR1 and PagATG8 could interact with each other, which is consistent with the interaction between the proteins of AtNBR1 and AtATG8 in *Arabidopsis*. These results suggested that the function is conserved between AtNBR1 and PagNBR1 (Figure 6). Thus, we hypothesized that the overexpression of *PagNBR1* may promote the degradation of the insoluble ubiquitinated protein. Therefore, the content of insoluble protein and ubiquitinated protein in insoluble protein was measured in WT and transgenic plant leaves under normal and salt stress conditions (Figures 7A–C). In Figure 7, the insoluble protein was observably enriched in WT compared with that in transgenic poplars under salt stress. However, no significant difference was found between WT and transgenic poplars under normal conditions (Figure 7A). The content of soluble protein also supported the above-mentioned results (Figure 7C). Meanwhile, the content of insoluble ubiquitinated protein was higher in WT than in transgenic plants. Furthermore, the content of insoluble protein and insoluble ubiquitinated protein was observably higher in the NaCl+ConA group than the NaCl group, the inhibition of autophagy resulted in the accumulation of insoluble protein and insoluble ubiquitinated proteins (Supplementary Figure S7). These results revealed that the overexpression of *PagNBR1* enhanced salt tolerance by promoting the degradation of insoluble ubiquitinated protein in transgenic poplars under salt stress.

Transcriptional levels of *ATG* genes have generally been used as an acceptable indicator to analyze the induction of autophagy under stress conditions in plants (Zhou et al., 2014; Chen et al., 2016; Sun et al., 2018a,b). To clarify whether autophagy is induced under salt stress, the transcriptional level of ATG3, ATG7, and ATG8 (a-g) genes were used to evaluate whether autophagy was induced under stress conditions (Figures 7H–J; Supplementary Figure S8) and the results showed that *ATG* genes (ATG8a-g, ATG3, and ATG7) increased to different degrees in WT and transgenic plants after salt stress compared with the control group. *ATG* gene expression levels in transgenic plants were observably higher than those in WT under salt stress. MDC has always been used as a marker of autophagosome in previous studies (Wang et al., 2015a). Our study showed that salt stress induced autophagy and that transgenic lines obtained higher autophagy activity under salt stress (Figures 7D,F). TEM is a valid and acceptable method used in plants for autophagosome detection (Sun et al., 2018a,b; Chi et al., 2020). To further investigate the induction of autophagy under salt stress conditions, TEM observation was performed, and the leaves of WT and transgenic plants under the control and treatment groups were used. Figures 7E,G show that the transgenic line accumulated more autophagosomes in the vacuole compared with WT lines after 4 days of treatment. Overall, these results demonstrated that overexpression of the *PagNBR1* gene enhanced tolerance of salt stress but induced the accumulation of autophagosomes in transgenic lines.

## Discussion

### NBR1 Is Evolutionarily Conserved in Eukaryotes

Selective autophagy is considered a specific process regulated by autophagy cargo receptors through interaction with ATG8 proteins (Jin et al., 2013). In mammals, the proteins of p62/SQSTM1 and NBR1-mediated selective autophagy targets ubiquitinated proteins and aggregate-prone proteins to degrade (Johansen and Lamark, 2011). The specific protein was also found because of the similar domain architectures and function with the metazoarl NBR1 and p62/SQSTM1 proteins (Svenning et al., 2011). The *PagNBR1* gene was cloned from Poplar 84 K. Analysis of the amino acid sequence revealed that the PagNBR1 protein was highly homologous with AtNBR1 protein and harbored the characteristic FW domain in NBR1 homologous proteins (Figure 1A and Supplementary Figure S1). The PB1-ZZ-UBA domain organizations harbored in mammal NBR1,p62/SQSTM1 and AtNBR1 were also found in the PagNBR1 protein. Meanwhile, the sub-cellular localization pattern of the PagNBR1 protein in tobacco protoplast (Figures 1B,C) and yeast two-hybrid analysis (Figure 6) revealed that the PagNBR1 protein was localized in autophagosomes and could interact with ATG8 proteins. These findings were consistent with AtNBR1 and suggest that PagNBR1 could be a candidate receptor of selective autophagy.

### *PagNBR1* Is a Crucial Candidate Gene for Breeding Salt-Tolerant Poplars

Autophagy contributes to the enhancement of tolerance of drought, salt, heat, and oxidative stress through degrading damaged proteins and organelles (Liu et al., 2009; Zhai et al., 2016). Previous studies have focused on the functional analysis of ATG genes, which are conserved genes for autophagosome formation in plants (Batoko et al., 2017). Our understanding of selective autophagy in response to abiotic stress remains limited. The AtNBR1 protein is the first selective autophagy receptor isolated from plants (Avin-Wittenberg, 2019). The *nbr1* mutant exhibits hypersensitivity to heat stress (Zhou et al., 2013), but its potential function in breeding stress-tolerant plants is unclear. Thus, we speculated that *PagNBR1* may be applied as a target gene for breeding stress-tolerant poplars. As the PagNBR1 is a candidate for the selective autophagy receptor, the increased expression levels of *PagNBR1* with leafage adding among young leaves, mature leaves and old leaves imply that it could play important roles in degrading and recycling damaged proteins and organelle during plant senescence. In addition, the expression levels of the *PagNBR1* gene were induced by stress-response plant hormones such as ABA, ET, SA, and Me-JA (Figures 2E–I), suggesting that the *PagNBR1* gene might be involved in stress tolerance. To further elucidate the potential breeding value in resisting salt stress and oxidative stress, we analyzed the expression pattern of the *PagNBR1* gene after treatment with NaCl, MV, or H_2_O_2_, and the transgenic poplar to estimate tolerance to salt stress. Our results showed that salt stress or oxidative stress increased the transcriptional level of the *PagNBR1* gene (Figures 2B–D). Under salt stress, *PagNBR1* overexpression in poplars exhibited enhanced capacity of salt stress than WT, and reduced damage to the cell membrane (Figures 3A–C), and maintained higher RWC (Figure 3D) and photosystem activity (Figures 3E–I). In addition, the FW/DW value of transgenic lines was higher than that of WT plants under salt stress but it was similar under normal conditions (Supplementary Figure S6). Therefore, *PagNBR1* overexpression conferred the tolerance of salt stress in transgenic poplars. In brief, *PagNBR1* is a crucial candidate gene for breeding salt-tolerant plants.

### *PagNBR1* Enhances Salt Stress Tolerance by Activating the Antioxidant System

Stress conditions always trigger the excessive accumulation of ROS, which leads to cell dysfunction and cell damage and death in plants (Raja et al., 2017; Yang and Guo, 2018). Thus, it is important for plants to maintain ROS homeostasis during stress conditions. In an apple, the overexpression of the *ATG18a* gene increases the activity of the antioxidant system, enhancing its tolerance to drought and alkaline stress (Sun et al., 2018a; Li et al., 2020). In *Arabidopsis*, heat stress triggered the accumulation of highly aggregate-prone proteins in *nbr1* mutant lines such as catalases, suggesting the important role of the NBR1 protein in regulating the antioxidant system (Zhou et al., 2014). Our results showed that *PagNBR1* overexpression in poplars reduced ROS levels by increasing enzyme activity including SOD, POD, CAT, transcriptional levels of genes involving ASA-GSH cycling (Figures 5C–L) and the content of AsA + DHA and GSH + GSSG (Supplementary Figure S6) contributed to relieving the damage of plant membranes, as evidenced by the results of MDA, electrolyte leakage, and higher photosynthetic capacity. These findings suggest that the *PagNBR1* protein enhanced salt stress tolerance by activating the oxygen scavenging system.

### *PagNBR1* Enhances Salt Stress Tolerance Through the Maintenance of K^+^/Na^+^ Homeostasis

**Figure 8:**
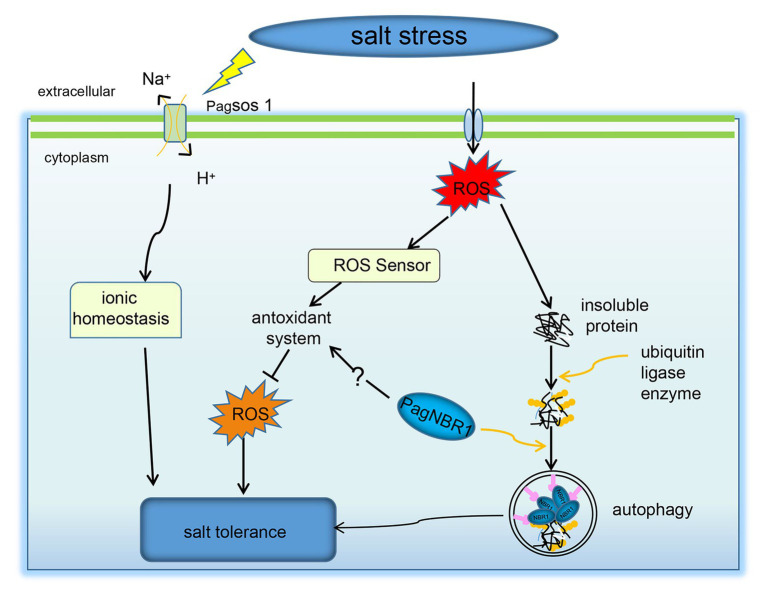
A possible model for NBR1-mediated stress resistance pathways. Under salt stress, the upregulated *PagNBR1* involved in the selective autophagy pathway facilitates the degradation of insoluble ubiquitinated protein, which contributes to the quality control of proteins. The upregulated *PagNBR1* may also mediate the activity of the antioxidant system, which maintains the homeostasis of ROS and K^+^/Na^+^ through the non-ubiquitinated pathway.

K^+^ is an important nutrient factor, and maintenance of a high K^+^/Na^+^ ratio is favorable for plant growth and development (Barragan et al., 2012; Almeida et al., 2017). However, under salt stress, Na^+^ accumulates in the cytoplasm, resulting in a decrease in K^+^/Na^+^. This phenomenon perturbs enzymatic functions and leads to plant cell damage (Almeida et al., 2017; Yang and Guo, 2018). Therefore, the maintenance of relatively high levels of the K^+^/Na^+^ ratio is important for enabling plants to resist salt stress. In our studies, the selective autophagy receptor PagNBR1 was verified to confer salt tolerance in transgenic lines. The transgenic poplars accumulated lower content of Na^+^ and maintained higher K^+^/Na^+^ than WT in leaves and roots under salt stress (Figures 4A–F). These results indicated the involvement of the *PagNBR1* gene in the redistribution of Na^+^ and K^+^. SOS1, which is a Na^+^/H^+^ antiporter, is responsible for extruding excessive Na^+^ from the cytoplasm to the extracellular environment (Yang et al., 2015). Research has shown that low levels of ROS increased the expression levels of *SOS1* and contributed to the maintenance of K^+^/Na^+^ homeostasis under salt stress (Wu et al., 2007; Chung et al., 2008). In our results, the lower levels of ROS in the transgenic line could act as a signal molecular, increasing the transcription level of *SOS1* (Supplementary Figure S3) and contributing to maintaining a higher K^+^/Na^+^ ratio under salt stress (Figure 8).

### *PagNBR1* Enhances Salt Stress Tolerance by Activating Autophagy

Excess ROS leads to proteins and organelles damage in plants, which is toxic to the survival of plants. Selective autophagy is considered to contribute to the degradation of insoluble ubiquitinated protein aggregates and organelles for recycling during imposed stress conditions (Johansen and Lamark, 2011; Avin-Wittenberg, 2019). In *Arabidopsis*, the sensitive phenotype characteristics of *nbr1* mutant in response to heat stress are related to the excessive accumulation of insoluble ubiquitinated protein aggregates (Zhou et al., 2013). Thus, we hypothesized that the increased tolerance of salt stress in transgenic poplars is associated with the degradation of insoluble ubiquitinated protein aggregates by inducing an autophagy system. In line with this hypothesis, the transgenic poplars accumulated less insoluble proteins and insoluble ubiquitinated proteins in contrast to WT lines under salt stress (Figures 7B,C). According to the results reported by Chen et al. (2016), the transcriptional levels of *ATG* genes and autophagic flux were used to investigate the involvement of autophagy in the submergence response and their results showed the upregulation of *ATG* genes associated with the increased autophagic flux, suggesting that the transcriptional levels of ATGs are an important indicator in investigating the induction of autophagy under stress conditions. The expression level of ATGs has been used as an important indicator to analyze the induction of autophagy under stress conditions in plants (Chen et al., 2016; Sun et al., 2018a,b). The results obtained from MDC-staining showed that autophagosomes were induced under salt stress and it accumulated more in transgenic lines than in WT, suggesting that autophagy was enhanced in the transgenic line compared with WT during salt stress (Figures 7D,F). In woody plants, such as apple, autophagy was well studied in terms of enhancing tolerance of stress conditions and the expression of *ATG* genes (eg., *ATG3, ATG5, ATG7, ATG8* et al.) have been widely used as an indicator for the induction of autophagy. It has been used together with the TEM to evaluate the autophagy activity in WT and transgenic lines (Sun et al., 2018a,b; Huo et al., 2020; Li et al., 2020). Thus, we detected the transcription level of ATG3, ATG7, and ATG8 (a-g) genes using the qRT-PCR method, and results (Figures 7H–G, Supplementary Figure S8) revealed that overexpression of the *PagNBR1* gene promoted the upregulation of ATG genes, suggesting that autophagy was induced under salt stress. Furthermore, our results revealed that transgenic poplars formed more vesicles in the vacuole in transgenic lines than that of WT lines (Figures 7E,G) which is similar to the previous results reported by Sun et al. (2018a,b), Huo et al. (2020), and Li et al. (2020) suggesting that overexpression of *PagNBR1* increased autophagy activity in transgenic lines under salt stress. Furthermore, inhibiting autophagy with ConA in WT and transgenic lines resulted in the similar accumulation of insoluble proteins and insoluble ubiquitinated proteins (Supplementary Figure S8) suggesting that the activated autophagy promoted the degradation of insoluble ubiquitinated proteins. In summary, the overexpression of *PagNBR1* confers the tolerance of salt stress in transgenic poplars through enhancing autophagy activity (Figure 8).

## Conclusion

Our results showed that PagNBR1 is a selective autophagy receptor that is homologous with AtNBR1. External environment factors such as salt stress, oxidative stress, and plant hormones (ABA, ET, SA, GA, me-JA) induced an increase in *PagNBR1*. Overexpression of *PagNBR1* in poplars enhanced the tolerance of salt stress *via* promoting degradation of insoluble ubiquinitated protein, which was associated with enhanced autophagy activity. In addition, the overexpression of *PagNBR1* enhanced the scavenging of ROS, which may be associated with maintaining higher K^+^/Na^+^ by increasing the expression levels of *SOS1*.

## Data Availability Statement

All datasets generated for this study are included in the article.

## Author Contributions

WS and XX conceived and designed the experiments. WS, YL, YB, SW, DW, and XY performed the experiments and analyzed the experimental data. WS wrote the article. WS, CL, WY, and XX revised the article. All authors contributed to the article and approved the submitted version.

### Conflict of Interest

The authors declare that the research was conducted in the absence of any commercial or financial relationships that could be construed as a potential conflict of interest.

## Footnotes

^1^https://www.ncbi.nlm.nih.gov/cdd

^2^http://web.expasy.org/protparam/

^3^http://www.rjkit.com/products.html

## Abbreviations

NBR1: neighbor of BRCA1 gene 1
ATG8: Autophagy-related gene 8
SOD: superoxide dismutase
CAT: Catalase
POD: Peroxidase and AsA, ascorbate
GSH: glutathione cycle
UBA: ubiquitin-associated
ROS: Reactive oxygen species
MDA: Malonaldehyde
EL: Electrolyte leakage
