# Supplementary material for: Poplar Autophagy Receptor NBR1 Enhances Salt Stress Tolerance by Regulating Selective Autophagy and Antioxidant System
Source: Front Plant Sci. 2021 Jan 20;11:568411. doi: 10.3389/fpls.2020.568411 (PMC7854912; doi:10.3389/fpls.2020.568411)
Supplement: Supplementary file 1 [file Table_1.docx]

 

Table S1 qRT-PCR, PagNBR1 CDS and selected primers used in this study

Gene name/ ID	Forword primer	Reverse primer
qRT-PCR
PagNBR1/Potri.012g085700	TGCGATGGATGTGGTGTTCA	CAAGACATCGGCCTGTCCAT
APX2/Potri.006G132200	GGTGCCATAAGGAGCGTTCT	TCAACAAGGGGACGGAAGAC
APX1/Potri.016G084800	ATTACTGGAGGGCCTGAGGT	CCACCGGATAGGGCAACAAT
DHAR1/Potri.008G049300	GGATTTGAGCTTGGCTCCGA	CATGCGTTGACCTTTGGCTC
DHAR2/Potri.004G00750	CGCTGCAATCTCTCCCTCAA	ATCTTTGAGCCCACAGAGGC
GR1/Potri.001G050000	TGGAAGCTGTAGGCGTTGAG	TCTGGTTTGGTAGGTTGCCC
GR2/Potri.003G178200	CTTCCATGTGTGGGCCAGAT	TAACACGCCTTGCCACTGAA
MDHAR/Potri.001G099600	GAAGCCAGTTCTGATGGGCA	ACCTGTATTCCGCCAACACT
SOS1/Potri.010G100900	TCCCGGTTTGTAGTTGTTGGATC	GAAGTACGATTCCACCAGTGAAG
ATG8a/Potri.004G013700	TGTGATTGTGGAAAGGGCTGA	CAATGAAGCGGTAGAAGGCAG
ATG8b/Potri.011G004300	TCTGCCTCCCACTGCTACAT	TCCAAAGGTATTCTCCCCGCT
ATG8c/Potri.002G228800	TGGAAAGGGCTGAGAAGAGTG	CATGGCAGCAGTAGGTGGTAG
ATG8d/Potri.014G153800	AGCTGGTCGCATCAGAGACA	CCTTTTCCGAACCACGTACAC
ATG8e/Potri.002G144600	CAATGTTCTCCCACCAACCG	GGCAACTCATGACACCCAAA
ATG8f/Potri.001G122700	CTCCCACCCACAGGTGCTAT	ACCCAAAAGTGTTCTCTCCGC
ATG8g/Potri.014G060300	AAGAGCCGTTTCAAGCAAGAG	ATCGCTTCTCTCAGCCTTCTC
ATG3/Potri.003G107000	GAGAATCACAGGTCCACGCA	GATTCCCAGGACCAAGTGGG
ATG7/Potri.004G055600	CCACTAGTAGCAGTGGTGGC	ACAACAGTGCTGCAACAAGC
PCR
PagNBR1/cds	ATGGATTCTACTATGGTCATCAAGG	AGCCTTCTCCCCGGTGAGAAT
Selected preimer
35S-GFP	CCGCAGCCATTAACGACTTT	AGCCTTCTCCCCGGTGAGAAT


Figure S1 The similar domain organizations contain in proteins of NBR1 and P62 in Homo sapiens, Saccoglossus kowalevskii, Strongylocentrotus purpuratus, Arabidopsis and Populus trichocarpa.
 . 


（B）（B）（A）（A）
Figure S2 Selection of transgenic plants. (A) PCR analysis of transgenic plants; (B) 
Relative expression level of PagNBR1 in selected lines by qRT-PCR. 


Figure S3 The relative expression level of SOS1 in wild-type (WT) and transgenic plants.


Figure S4 phenotypic analysis of PagNBR1 overexpression lines under 200 mM NaCl for 10 days.

Figure S5 FW/DW of shoots and roots in WT and transgenic line at the end of treatment.These datas were measured after 4 days of treatment. Values are means (± SE) of three independent experiments (n=3). Data were analyzed through LSD multiple range tests in the ANOVA program of SPSS (IBM SPSS17.0).Different letters indicate significantly differences bteween treatment at level of P < 0.05.

Figure S6 Content of AsA+DHA and GSH+GSSG in WT and transgenic lines at the end of the treatment.These datas were measured after 4 days of treatment. Values are means (± SE) of three independent experiments (n=3). Data were analyzed through LSD multiple range tests in the ANOVA program of SPSS (IBM SPSS17.0).Different letters indicate significantly differences bteween treatment at level of P < 0.05.

Figure S7 Rlitive Content ofinsouble proteins and insouble Ubiquinitated proteins in WT and transgenic lines at the end of the treatment. These datas were measured after 6h of treatment using detached leaf under 200mMNaCl or 200mM NaCl+0.5uMConA. All treatment repeat three times. Data were analyzed through LSD multiple range tests in the ANOVA program of SPSS (IBM SPSS17.0).Different letters indicate significantly differences bteween treatment at level of P < 0.05.

Figure S8 Relative expression levels of ATG8b-g in WT and transgenic poplars under control and salt stress. These datas were measured after 4 days of treatment. Values are means (± SE) of three independent experiments (n=3). Data were analyzed through LSD multiple range tests in the ANOVA program of SPSS (IBM SPSS17.0).Different letters indicate significantly differences bteween treatment at level of P < 0.05.


Supplement methods 
Genetic transformation of PagNBR1 gene
The transgenic poplars is generated by the Agrobacterium-mediated transformation. In brief, the leaf discs of poplar 84K were infected with the Agrobacterium EHA105. Then, the infected leaf discs were transferred to the co-culture medium（MS+ 0.50 mg/L 6- benzylaminopurine (6-BA)+0.020 mg/L thidiazuron (TDZ)+100mM/lAS+30g/Lsugar+8g/Lagar for 3days. After that, the leaf discs were transferred to inducing-medium(（MS + 0.50 mg/L 6- benzylaminopurine (6-BA)+0.020 mg/L thidiazuron (TDZ) +250 mg/L cefotaxime +4 mg/L hygromycin + 30g/L sugar + 8g/L agar) to produce callus and shoots. The shoots were transfered to the rooting medium. Hygromycin was used to screen the positive lines since the callus-inducing stage. Finally, the screened seedlings were performed to the PCR with the primer sequences designed from promoter (forward primer) and NBR1 (reverse primer). qRT-PCR were also used to analyze the expression level of NBR1 gene in the screened positive lines. 

Treatment of salt stress
The WT and transgenic lines were placed in the trays and added enough water in it. For treatment group 400 mM NaCl solution (3.5L) were added in the trays to replace the water. For control group, WT and transgenic lines were cultured in the same condition but replace the NaCl solution with water. The phenotypes were observed every 24h, ill the emergence of the damaged spots exhibited on the leaves of plants in the treatment group. After treatment the materials were collected to detecte the Physiological indicators.
